# Impact of Living Place on the Prognosis of Aspiration Pneumonia in Elderly Patients: A Retrospective Study in Tokyo

**DOI:** 10.7759/cureus.98407

**Published:** 2025-12-03

**Authors:** Yuki Shimazu, Clara So, Torahiko Jinta, Tomoaki Nakamura, Kohei Okafuji, Atsushi Kitamura, Yutaka Tomishima, Naoki Nishimura

**Affiliations:** 1 Department of Internal Medicine, St. Luke's International Hospital, Tokyo, JPN; 2 Department of Pulmonary Medicine, Thoracic Center, St. Luke's International Hospital, Tokyo, JPN

**Keywords:** aspiration pneumonia, geriatrics, nursing home, prognosis, pulmonology

## Abstract

Introduction

In Japan, aspiration pneumonia is a major cause of death among the elderly. While clinical factors are well-documented, the impact of social factors on the prognosis of aspiration pneumonia remains underexplored. This study investigates whether social factors, such as living place and caregiver presence, influence the prognosis of aspiration pneumonia.

Methods

This retrospective study analyzed patients aged 65 and older admitted for aspiration pneumonia at our hospital from January 2012 to December 2022. The study included 650 patients after excluding those with pneumonia onset after admission, interstitial pneumonia, and cancer or those on immunosuppressants. Clinical data, including age, sex, comorbidities, and Pneumonia Patient Outcomes Research Team (PORT) score, were collected alongside social factors such as the level of care needed, living place, and cohabitant number at home. We performed univariate and multivariable analyses to assess the relationship between these factors and mortality rates.

Results

The overall mortality rate was 18.6%. Significant differences were found in age, sex, congestive heart failure, and PORT score between survivors and non-survivors. Multivariable analysis revealed that residing in a nursing home is associated with a lower mortality rate than living at home (odds ratio, 0.62; 95% CI, 0.40-0.95). On the other hand, patients living at home with two or more cohabitants had a higher mortality rate than those in nursing homes, although this was not statistically significant (odds ratio, 1.67; 95% CI, 0.94-2.94).

Discussion

The study indicates that residing in a nursing home may improve the prognosis of aspiration pneumonia. Patients at home may experience delayed medical attention and less effective preventive measures than those in nursing homes.

Conclusion

Social factors, particularly the patient's living place, impact the prognosis of aspiration pneumonia. Enhanced caregiving in a nursing home may contribute to a better prognosis, emphasizing the need for improved home care strategies.

## Introduction

In Japan, which has an aged society, aspiration pneumonia is a major cause of death [1]. Aspiration pneumonia significantly affects the lifespan of the elderly. In fact, the one-month mortality rate of aspiration pneumonia is 17.2% [2], and the three-month mortality rate of it is 38.6% [3]. Therefore, predicting the prognosis is important for both patients and their families. The cause of aspiration pneumonia is associated with aspiration episodes, so not only clinical factors but also social factors, such as the patient's level of independence in daily living, where the patient lives, and who the patient's caregiver is, might be related to the prognosis of aspiration pneumonia.

Previous studies have identified age, sex, comorbidities, the severity of dysphagia, the level of daily living independence, nutritional status, the presence of alternative nutrition, and oral hygiene as prognostic factors for aspiration pneumonia [4-9]. To the best of our knowledge, few studies have investigated the social factors that impact the prognosis of aspiration pneumonia.

According to Tomonaga et al.'s study, half of aspiration pneumonia cases occur at home, and half occur in nursing homes [10]. This study surveyed social factors such as the patient's living place and who the patient's caregiver is. Many patients with aspiration pneumonia are elderly, so caregiving to prevent aspiration is crucial for aspiration pneumonia. It is highly possible that the prognosis differs between patients with well-established social services and those without.

This study aims to investigate whether social factors are associated with the prognosis of aspiration pneumonia and to understand how we can adjust for social factors to improve the prognosis of aspiration pneumonia.

## Materials and methods

Subjects

This retrospective study was conducted targeting patients aged 65 years and older who were admitted to the Department of Pulmonary Medicine or General Medicine at our hospital for aspiration pneumonia between January 2012 and December 2022. The Institutional Review Board of St. Luke's International Hospital issued approval 23-J007. The diagnostic criteria for aspiration pneumonia were based on the diagnostic flowchart for aspiration pneumonia described in the Japanese Guidelines for the Management of Nursing- and Healthcare-Associated Pneumonia. Specifically, aspiration pneumonia was diagnosed in patients who showed radiographic evidence of pneumonia on chest X-ray or chest CT and had elevated C-reactive protein (CRP) levels, together with either witnessed aspiration or a suspected decline in swallowing function [11].

Exclusion criteria included the onset of aspiration pneumonia after hospitalization, concurrent interstitial pneumonia, concurrent neoplastic disease requiring treatment, and the ongoing administration of immunosuppressive agents.

Data collection

We set mortality as the primary outcome. Data on clinical factors such as age, sex, comorbidities (neoplastic disease, liver disease, cerebrovascular disorder, congestive heart failure, and kidney disease), and Pneumonia Patient Outcomes Research Team (PORT) score were extracted [12]. The PORT score, which is used as a prognostic score for community-acquired pneumonia, includes a wide range of prognostic factors, such as comorbidities and vital signs at admission. The PORT score includes clinical factors so broadly that using the PORT score as an independent variable is useful when considering the influence of only social factors in multivariable analysis.

The level of care needed, where the patient lives, and who the patient's caregiver is were extracted as social factors. In Japan, the long-term care system is structured to provide support to individuals who need assistance due to aging, illness, or disability. The system includes different levels of care: support level 1 or 2 and care levels 1-5. Table 1 shows that each level signifies a different degree of care needed [13]. We categorized the patients' living place as either living at home or residing in a nursing home. In the case of the patients who live at home, the numbers of their cohabitants were also extracted.

**Table 1:** Classification of the level of care needed The content is summarized by the authors based on publicly available information from the Ministry of Health, Labour and Welfare in Japan [13]

Level of care needed	Definition
Support level 1	Individuals at this level require some assistance with daily activities but are relatively independent. They might need help with complex tasks such as meal preparation and cleaning but can manage personal care independently.
Support level 2	These individuals need more assistance than those in support level 1. They require help with both personal care and household tasks more frequently but can still maintain some level of independence.
Care level 1	Individuals need partial assistance with daily activities such as bathing, dressing, and eating. They may also require support with mobility and other basic personal care tasks.
Care level 2	At this level, individuals require a higher degree of support, including significant help with personal care and some medical supervision. They often need assistance with most daily activities.
Care level 3	Individuals need extensive assistance with daily activities and regular supervision. They require help with most personal care tasks and some medical needs. This level typically indicates a moderate degree of dependency.
Care level 4	These individuals need intensive assistance with almost all daily activities and constant supervision. They require significant help with personal care and medical needs and may have limited mobility.
Care level 5	The highest level of care, individuals at this level are almost entirely dependent on caregivers for all aspects of daily living. They need comprehensive support with personal care, medical care, and constant supervision.

Statistical analyses

A univariate analysis was performed using t-tests and chi-square tests, and a multivariable analysis was performed using logistic regression analysis. In the multivariable analysis, the "nursing home resident" item of the PORT score was confounded with the independent variable of whether the patient resides in a nursing home or at home. Therefore, we used the modified PORT score, which excludes the "nursing home resident" item of the PORT score, for the multivariable analysis. We utilized the patient's living place, the level of care needed, and the number of cohabitants at home as independent variables representing social factors. The number of cohabitants at home was treated as a categorical variable, classified into either "one or zero" or "two or more."

The significance level was set at P < 0.05. All statistical analyses were conducted using R (R Foundation for Statistical Computing, Vienna, Austria) and RStudio (Posit Software, Boston, MA) software.

## Results

**Figure 1:**
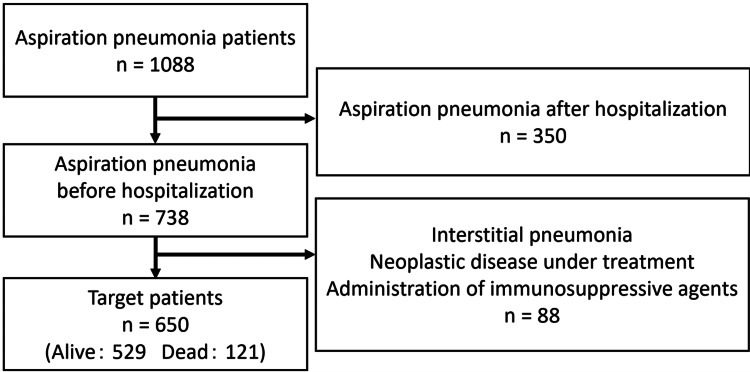
Flowchart of patient selection

Figure 1 illustrates the flowchart for selecting the study participants. Among 1088 patients who met the diagnostic criteria for aspiration pneumonia, 350 developed aspiration pneumonia after admission. A total of 88 patients had concurrent interstitial pneumonia, ongoing treatment for malignant tumors, or immunosuppressive agents. Thus, the analysis included 650 patients, 529 survivors and 121 deceased patients, resulting in a mortality rate of 18.6%.

The patients' clinical characteristics are presented in Table 2. Age, sex, the presence of congestive heart failure, and PORT score showed significant differences. Conversely, no significant differences were observed in social factors, such as the level of care needed (classified as no care application or applied but unclear/support level 1 to care level 3/care level 4 to care level 5) and living environment (nursing home/home) (Table 3). Table 4 compares the characteristics of patients in the nursing home group and the home group. Patients in the nursing home group were older, had a higher PORT score at admission, and more often had a higher level of care need. However, no significant difference in mortality was observed between the two groups. A multivariable analysis using a modified PORT score and social factors as independent variables revealed significant differences, with lower mortality rates observed in the nursing home group than in the home group (Table 5) (odds ratio, 0.62; 95% CI, 0.40-0.95).

**Table 2:** Clinical characteristics of the patients PORT: Pneumonia Patient Outcomes Research Team

Characteristics	Total (n = 650)	Alive (n = 529)	Dead (n = 121)	T value or chi value	P value
Age (years, mean ± SD)	86.7 ± 7.5	86.3 ± 7.7	88.2 ± 6.5	-2.69	0.016
Sex					
Male (%)	334 (51.4)	261 (49.3)	73 (60.3)	4.33	0.037
Female (%)	316 (48.6)	268 (50.7)	48 (39.7)		
Comorbidity					
Neoplastic disease (%)	129 (19.8)	103 (19.5)	26 (21.5)	0.14	0.71
Liver disease (%)	22 (3.4)	18 (3.4)	4 (3.3)	0.4 × 10^-28^	1.0
Cerebrovascular disease (%)	255 (39.2)	216 (40.8)	39 (32.2)	2.71	0.10
Congestive heart failure (%)	115 (17.7)	81 (15.3)	34 (28.1)	10.2	<0.001
Renal disease (%)	80 (12.3)	59 (11.2)	21 (17.4)	2.96	0.085
PORT score (mean ± SD)	140 ± 26.9	137 ± 26.6	153 ± 24.7	-6.055	<0.001

**Table 3:** Social characteristics of the patients

Characteristics	Total (n = 650)	Alive (n = 529)	Dead (n = 121)	T value or chi value	P value
Level of care needed					
No care application or applied but unclear (%)	152 (23.4)	125 (23.6)	27 (22.3)	0.036	0.85
Support level 1 to care level 3 (%)	206 (31.7)	165 (31.2)	41 (33.9)	0.22	0.64
Care level 4 to care level 5 (%)	292 (44.9)	239 (45.2)	53 (43.8)	0.030	0.86
Living place					
Nursing home (%)	264 (40.6)	223 (42.2)	41 (33.9)	2.46	0.12
Home (%)	386 (59.4)	306 (57.8)	80 (66.1)		
The number of cohabitants at home					
Zero cohabitant (%)	60 (9.2)	46 (7.1)	14 (2.2)	0.66	0.42
One or more cohabitants (%)	326 (50.2)	260 (40.0)	66 (10.2)	0.94	0.33
Zero or one cohabitant (%)	262 (40.3)	208 (39.3)	54 (44.6)	0.94	0.33
Two or more cohabitants (%)	124 (19.1)	98 (18.5)	26 (21.5)	0.38	0.54

**Table 4:** Comparison of characteristics between the nursing home group and the home group PORT: Pneumonia Patient Outcomes Research Team

Characteristics	Total (n = 650)	Home (n = 386)	Nursing home (n = 264)	T value or chi value	P value
Age (years, mean ± SD)	86.7 ± 7.5	85.8 ± 7.6	88.0 ± 7.1	-3.82	<0.001
Sex					
Male (%)	334 (51.4)	222 (57.5)	112 (42.4)	13.7	<0.001
Female (%)	316 (48.6)	164 (42.5)	152 (57.6)		
Comorbidity					
Neoplastic disease (%)	129 (19.8)	75 (19.4)	54 (20.5)	0.049	0.83
Liver disease (%)	22 (3.4)	15 (3.9)	7 (2.7)	0.40	0.53
Cerebrovascular disease (%)	255 (39.2)	141 (36.5)	114 (43.2)	2.64	0.10
Congestive heart failure (%)	115 (17.7)	72 (18.7)	43 (16.3)	0.45	0.50
Renal disease (%)	80 (12.3)	49 (12.7)	31 (11.7)	0.058	0.81
PORT score (mean ± SD)	140 ± 26.9	134 ± 26.0	149 ± 25.9	-7.07	<0.001
Level of care needed					
No care application or applied but unclear (%)	152 (23.4)	101 (26.2)	51 (19.3)	3.73	0.05
Support level 1 to care level 3 (%)	206 (31.7)	140 (36.3)	66 (25.0)	8.68	0.003
Care level 4 to care level 5 (%)	292 (44.9)	145 (37.6)	147 (55.7)	20.07	<0.001
Mortality	121 (18.6)	80 (20.7)	41 (15.5)	2.46	0.12

**Table 5:** Multivariable analysis of modified PORT score and social factors predicting mortality in patients with aspiration pneumonia Modified PORT score: PORT score that excludes the "nursing home resident" item PORT: Pneumonia Patient Outcomes Research Team

	Odds ratio (95% CI)	P value
Modified PORT score	1.03 (1.02-1.03)	<0.001
Level of care needed		
No care application or applied but unclear/care level 4 to care level 5	1.04 (0.61-1.77)	0.87
Support level 1 to care level 3/care level 4 to care level 5	1.05 (0.65-1.68)	0.85
Living place		
Nursing home/home	0.62 (0.40-0.95)	0.030

Furthermore, the multivariable analysis categorizing the home group based on the number of cohabitants showed that even patients residing at home with two or more cohabitants tended to have a higher mortality rate compared to those in nursing homes, although there was no significant difference (Table 6) (odds ratio, 1.67; 95% CI, 0.94-2.94).

**Table 6:** Multivariable analysis of the modified PORT score and social factors focusing on the number of cohabitants predicting mortality in patients with aspiration pneumonia PORT: Pneumonia Patient Outcomes Research Team

	Odds ratio (95% CI)	P value
Modified PORT score	1.03 (1.02-1.03)	<0.001
Level of care needed		
No care application or applied but unclear/care level 4 to care level 5	1.05 (0.61-1.78)	0.87
Support level 1 to care level 3/care level 4 to care level 5	1.05 (0.65-1.69)	0.84
Living place and the number of cohabitants at home		
Zero or one cohabitant/nursing home	1.60 (1.00-2.58)	0.053
Two or more cohabitants/nursing home	1.67 (0.94-2.94)	0.076

## Discussion

This study highlights that the living place of elderly patients is a significant social determinant of mortality in aspiration pneumonia, with an overall mortality rate of 18.6%. Aspiration pneumonia primarily affects elderly individuals, largely due to their multiple comorbidities and repeated episodes of aspiration resulting from a decline in swallowing function [14]. Furthermore, a multivariable analysis considering a wide range of clinical factors and social factors showed that the level of care needed and the number of the patient's cohabitants at home did not significantly affect the prognosis of aspiration pneumonia, whereas the patient's living place did.

This study is the first to suggest the potential influence of the patient's living place on the prognosis of aspiration pneumonia. The lower mortality rates observed among patients in nursing homes compared to those at home may be due to better nutritional status in nursing homes [14]. At home, preparing meals adapted to the patient's swallowing ability and assisting with their meals can be burdensome for families. In contrast, nursing homes can consistently provide well-balanced, dysphagia-friendly meals, with staff available to assist during mealtime. These nutritional advantages may contribute to greater physical resilience against aspiration pneumonia.

Another factor may be oral hygiene, which tends to be better maintained in nursing homes [15]. Poor oral hygiene can lead to increased oral bacterial growth, especially in elderly individuals who produce less saliva. Patients with weakened swallowing and cough reflexes are particularly susceptible to aspirating these bacteria, increasing their risk of infection. In institutional settings, oral care practices are typically routine and supervised, potentially lowering the bacterial burden that contributes to aspiration pneumonia.

Additionally, caregivers in nursing homes are generally more familiar with preventive strategies for aspiration [16], including monitoring patients' eating pace and posture [17]. These factors are critical in preventing aspiration events. In contrast, at home, family members may not recognize the early signs of aspiration pneumonia, leading to delays in seeking medical care. Prompt recognition and early intervention can significantly improve outcomes, highlighting the importance of trained caregivers and structured care environments.

The limitations of this study include its retrospective nature in a single institution and the lack of outcome tracking after discharge. As discussed above, this study proposes possible mechanisms, such as differences in nutritional management, oral hygiene, and caregiver expertise, to explain the better prognosis observed in nursing homes. However, these factors were not directly measured in our analysis. Therefore, the proposed explanations remain speculative and should be explored in future prospective studies that incorporate these variables. Future research should also examine how differences in caregiving services among nursing homes, or the frequency of home care and nursing visits, affect the prognosis of aspiration pneumonia.

## Conclusions

This study demonstrates that the living place, specifically residing in a nursing home, is associated with a lower mortality rate in elderly patients with aspiration pneumonia, whereas other social factors, such as the level of care needed and the number of cohabitants at home, do not significantly impact prognosis.

The findings suggest that the comprehensive care provided in a nursing home may contribute to improved outcomes. Recognizing the importance of living place as a prognostic factor could help inform care strategies and early intervention practices to reduce mortality in patients with aspiration pneumonia.
